# Paucatalinone A from Paulownia Catalpifolia Gong Tong Elicits mitochondrial-mediated cancer cell death to combat osteosarcoma

**DOI:** 10.3389/fphar.2024.1367316

**Published:** 2024-03-25

**Authors:** Ganyu Wang, Zhiwei Cui, Jinqiu Tian, Xinyuan Li, Wenzhao Tang, Weiqiang Jing, Aiwu Li, Yuankai Zhang

**Affiliations:** ^1^ Department of Pediatric Surgery, Qilu Hospital, Cheeloo College of Medicine, Shandong University, Jinan, Shandong Province, China; ^2^ Department of Orthopedics, Qilu Hospital, Cheeloo College of Medicine, Shandong University, Jinan, Shandong Province, China; ^3^ Department of Immunology, Shandong Provincial Key Laboratory of Infection Immunology, School of Basic Medical Sciences, Cheeloo College of Medicine, Shandong University, Jinan, Shandong Province, China; ^4^ School of Parmacy and Pharmaceutical Sciences, Institute of Materia Medica, Shandong First Medical University, Shandong Academy of Medical Sciences, NHC Key Laboratory of Biotechnology Drugs (Shandong Academy of Medical Sciences), Key Lab for Rare Uncommon Diseases of Shandong Province, Jinan, China; ^5^ Department of Urology, Qilu Hospital, Cheeloo College of Medicine, Shandong University, Jinan, Shandong Province, China

**Keywords:** osteosarcoma, Paucatalinone A, Paulownia catalpifolia Gong Tong, mitochondria, cell apoptosis

## Abstract

As the global cancer burden escalates, the search for alternative therapies becomes increasingly vital. Natural products, particularly plant-derived compounds, have emerged as promising alternatives to conventional cancer treatments due to their diverse bioactivities and favorable biosafety profiles. Here, we investigate Paucatalinone A, a newly discovered geranylated flavanone derived from the fruit of *Paulownia Catalpifolia* Gong Tong, notable for its significant anti-cancer properties. We revealed the capability of Paucatalinone A to induce apoptosis in osteosarcoma cells and deciphered its underlying mechanisms. Our findings demonstrate that Paucatalinone A substantially augments apoptosis, inhibits cell proliferation, and demonstrates a pronounced anti-tumor effect in a murine model of osteosarcoma. Mechanistically, Paucatalinone A disrupts calcium homeostasis and exacerbates intracellular reactive oxygen species accumulation, leading to mitochondrial impairment, cytoskeletal collapse, and caspase-dependent apoptotic cell death. This study underscores the potential of Paucatalinone A in initiating apoptosis in cancer cells and highlights the therapeutic efficacy of plant-derived agents in treating osteosarcoma, offering a viable approach for managing other intractable cancers.

## 1 Introduction

The increasing incidence of cancer across the globe has intensified the urgency for innovative and effective therapeutic strategies. As the second leading cause of death worldwide, cancer presents a multifaceted challenge that intertwines with the complexities of disease progression, treatment resistance, and diverse patient responses (Siegel et al., 2022). Among various cancer types, osteosarcoma stands out as a particularly aggressive form of malignant bone tumor. Predominantly affecting children and young adults aged 10 to 30, with a peak incidence during adolescent growth, osteosarcoma represents a critical area of oncological research (Ritter and Bielack, 2010). The disease’s severity is further underscored by the fact that approximately 10%–15% of newly diagnosed patients present with metastatic foci, primarily in the lungs. This metastatic nature significantly complicates treatment and prognosis. The current standard care for osteosarcoma includes surgical resection of the primary tumor, supplemented by neoadjuvant chemotherapy (Meltzer and Helman, 2021). Despite significant advancements in these treatments, the emergence of resistance to chemotherapy and a consequent rise in mortality rates have raised serious concerns in the medical community (Isakoff et al., 2015). This situation highlights an urgent need for novel, more effective anticancer agents that can overcome such resistance and improve patient outcomes.

Historically, plants have been a cornerstone in medicinal treatments, with their use dating back to the origins of human civilization (Atanasov et al., 2015; Agarwal et al., 2020). In the realm of cancer therapy, the exploration of plant-based compounds has gained momentum due to their potential in providing safer, cost-effective, and efficacious alternatives to conventional treatments. The limitations associated with modern chemotherapy, such as high expenses and severe adverse effects, have only served to amplify the appeal of phytochemicals in cancer research (Cragg and Newman, 2005; Zhu et al., 2018; Yuan et al., 2020; Anywar et al., 2021). These plant-derived constituents offer a promising avenue for the development of new therapeutic agents, as evidenced by the fact that approximately 40% of treatments approved by the U.S. Food and Drug Administration (FDA) have their origins in natural sources (Newman and Cragg, 2012).

**FIGURE 1:**
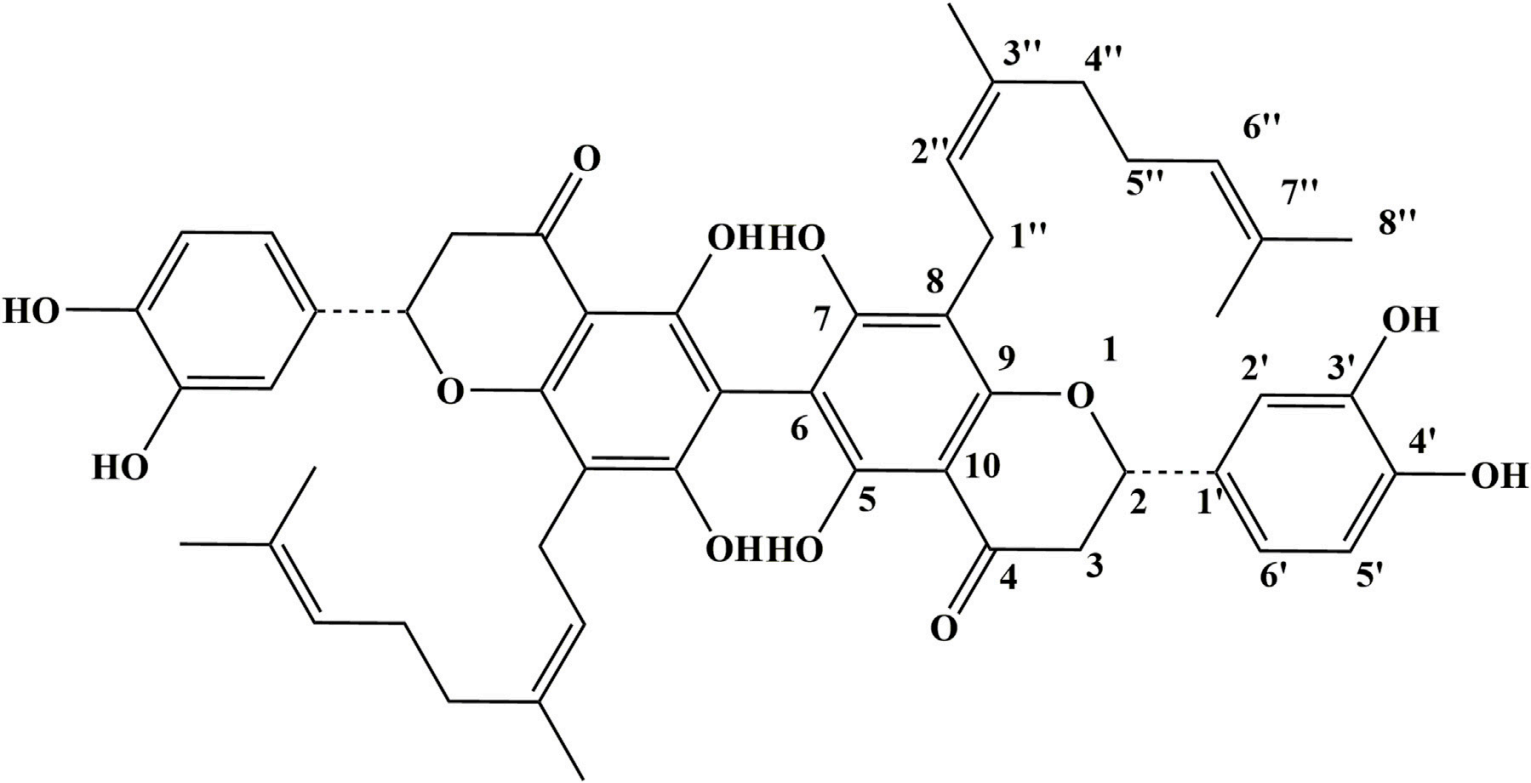
Structure of Paucatalinone A.

In this study, we focus on Paucatalinone A, a novel phytochemical from the perennial deciduous tree Paulownia catalpifolia Gong Tong (Figure 1A). Our prior investigation first isolated this compound from the fruits of Paulownia catalpifolia Gong Tong and had revealed the potential of Paucatalinone A in exerting bioactivity against human lung cancer cell line (Gao et al., 2015). This finding set the stage for a deeper exploration of its therapeutic efficacy and mechanism of action, particularly in the context of osteosarcoma.

Based on our own preliminary results and observation, we hypothesized that Paucatalinone A could promote tumor cell death by cell-autonomous mechanisms independently and develop as a potential therapeutic agent for malignancies. Here, we found that Paucatalinone A could regulate the calcium signaling pathway to break down the calcium homeostasis, generate excessive reactive oxygen species (ROS), induce cellular physiological activities disorders and collapse the cytoskeleton of osteosarcoma cells, thus achieving safe and efficient cancer therapy. What’s more, we further confirmed the same potency in osteosarcoma murine models. In short, our study would provide Paucatalinone A as an antitumor biotherapeutic candidate, which may have better clinically acceptable safety for cancer therapy.

## 2 Methods

### 2.1 Compound

Paucatalinone A was synthesized and analyzed by our project team member (Prof. Wenzhao Tang), as previously reported (Gao et al., 2015). Paucatalinone A was solubilized in DMSO and diluted according to different concentrations using complete medium for further cell study.

### 2.2 Cell culture

HOS and MG63 human osteosarcoma cell lines were obtained from American Type Culture Collection (ATCC), and cultured in Eagle’s Minimum Essential Medium (EMEM) supplemented with 10% fetal bovine serum (FBS), 100 μg/mL penicillin, 100 U/mL streptomycin. Mouse osteosarcoma cell line K7M2 was maintained in complete Dulbecco’s Modified Eagle’s Medium (DMEM). All cell lines were grown in incubator at 37°C with 5% CO_2_. Cells were seeded in culture dish and treated with different dose of Paucatalinone A for indicated times. For cell morphology examination, bright-field images of apoptotic cells were captured.

### 2.3 Cell proliferation assay and colony formation assay

The proliferation of cells was evaluated by sulforhodamine B (SRB) colorimetric assay and colony formation assay. For SRB assay, cells were seeded onto 96-well culture plates at a density of 3 × 10^3^ cells/well and incubated overnight. The next day, cells were treated with indicated concentration of Paucatalinone A for 24–72 h, with the indicated concentration of DMSO as control vehicle. After dissolving in Tris solution, the results were measured using microtiter plate reader at 560 nm (Vichai and Kirtikara, 2006). For colony formation assay, cells (1 × 10^3^) were plated onto culture dishes (35 mm) and cultured for 24 h. Then the media were replaced with drug-containing media and incubated for 48 h. After drugs treating, cells were cultured in fresh medium for another 12 days. Cell colonies were then fixed and stained with 0.01% crystal violet, followed with images capture.

### 2.4 Cell cycle analysis

Briefly, osteosarcoma cells (1 × 10^5^) were seeded onto 6-well plates, incubated overnight, and treated with drugs in medium for 48 h. After that, cells were collected, washed with PBS, fixed with ice-cold 70% ethanol, and kept at −20°C for at least 2 h. After washing twice with PBS, cells were incubated with 200 μg/mL RNase for 30 min at room temperature. Then cells were incubated with 50 μg/mL PI at 4°C for 30 min. The percentages of cells at each phase of cell cycle were measured by flow cytometry with 1 × 10^4^ events recorded for each sample. And the results were analyzed using FlowJo software.

### 2.5 Spheroid formation assay

To produce hanging drops, 15–30 μL cell suspension solution (containing 1.2% methyl cellulose) with cell number around 1000 cells per drop is pipetted on the lid of the 96-well plate. To prevent water evaporation, we added each well with PBS, and the lid was inverted over the culture plate. We utilized two types of stably transfected cells expressing fluorescent proteins, MG63-GFP and K7M2-GFP, to generate spheroid (Friedrich et al., 2009; Zhao et al., 2019). After 5-day incubation under standard condition (37°C with 5% CO_2_), the spheroids were harvested and imaged under the confocal microscopy at 488 nm wavelength.

### 2.6 Cell death assay

Cell death was measured using Annexin Ⅴ-FITC/propidium iodide (PI) cell apoptosis detection kit (BD Biosciences, cat: 556547) according to the manufacturer’s directions. Briefly, osteosarcoma cells (1 × 10^5^) were seeded onto 6-well plates, incubated overnight, and treated with drugs in medium for 72 h. After that, cells were collected and washed twice with PBS through centrifugation. Then cells were re-suspended in 100 μL binding buffer. Next, cells were stained with FITC-conjugated Annexin Ⅴ and PI according to the protocol, and incubated for 10 min at room temperature in the dark. Thereafter, the samples were analyzed using flow cytometry with 1 × 10^4^ events recorded for each sample and calculated for apoptotic rates, and the results were analyzed using CytExpert software. PI is excited by 488 nm wavelength and emitted by 585 nm wavelength. Annexin Ⅴ-FITC is excited by 488 nm wavelength and emitted by 525 nm wavelength.

### 2.7 Wound-healing scratch assay

Briefly, osteosarcoma cells (3 × 10^5^) were seeded on 6-well culture plates. After cells reaching confluence, the artificial wounds were created by scraping the confluent monolayer cells with a sterile 200 μL pipette tip. After washing, cells were treated differently. Pictures were captured by phase-contrast microscopy at 0, 24, 48 h after scratching.

### 2.8 Microfilament structure staining

For fluorescence imaging, osteosarcoma cells (1 × 10^5^) were seeded on the glass coverslips (24 mm) in 6-well culture plates. After 48 h of Paucatalinone A treatment, the glass coverslips were washed with PBS, then fixed with 4% formaldehyde. 0.5% Triton-X-100% and 0.25% BSA was used for cell permeabilization and blocking, respectively. The TRITC-phalloidin (MeilunBio, cat: MB5936, Ex = 540–546 nm, Em = 565–575 nm) was added and incubated for 2 h at room temperature. After washing, cells were stained with DAPI and mounted on a slide using anti-fade mountant. Fluorescent images were acquired using a confocal spinning-disk microscope (Andor, Dragonfly 200) at 561 nm (TRITC-phalloidin) and 405 nm (DAPI) wavelength, and analyzed using Imaris software.

### 2.9 Tom-20 staining

For visualization of mitochondrial structure, the osteosarcoma cells (1 × 10^5^) were seeded on the glass coverslips (24 mm) in 6-well culture plates, then treated with different drugs for 48 h. Cells were fixed with 4% paraformaldehyde for 30 min and permeabilized with 0.5% Triton X-100 for 10 min. Then cells were incubated with Tom-20 antibodies (Cell Signaling Technology, cat: 42406) at 4°C overnight. After washed with cold PBS for three times, cells were incubated with the second antibody. The nuclei were stained with DAPI. Images were captured with a confocal spinning-disk microscope (Andor, Dragonfly 200) at 488 nm (Tom-20) and 405 nm (DAPI) wavelength, and analyzed using Imaris software.

### 2.10 Ki67 staining

For fluorescence imaging, the tissue sections were deparaffinized by xylene, dehydrated in ethanol, and boiling for antigen retrieval. After blocking, the sections were incubated with anti-Ki67 antibody (Cell Signaling Technology, cat: 9129S) and the plasma membrane probe WGA Lectin (GeneTex, cat: GTX01502) at 4°C. The confocal microscopy (Andor, Dragonfly 200) was used to capture the images at 561 nm (Ki67) and 488 nm (WGA) wavelength.

### 2.11 TUNEL staining

TUNEL Apoptosis Assay Kit (Beyotime Biotechnology, cat: C1090) was used for DNA fragmentation and cell apoptosis detection. Briefly, the sections were treated with TdT enzyme, followed with DAPI staining for nuclei. Fluorescent images were detected by visualization with a confocal microscopy (Andor, Dragonfly 200) at 561 nm (TUNEL) and 405 nm (DAPI) wavelength.

### 2.12 Histology

At the animal experimental end point, the osteosarcoma tumors and main organs were collected for further analysis. After fixation, the tissues were embedded in paraffin, sliced into sections and histologically examined following hematoxylin and eosin (H&E) staining. The images were acquired using a VS120 Olympus microscope with OlyVIA software (Olympus).

### 2.13 Western blot

Western blot analysis was performed following standard methods. Briefly, osteosarcoma cells (3 × 10^5^) were seeded on 6-well culture plates. After corresponding Paucatalinone A treatment for 24 h, cells were washed twice and then lysed in lysis buffer supplemented with protease inhibitor cocktail. After protein concentration determination using BCA protein assay kit, cell extracts were mixed with 6× SDS loading buffer, followed by SDS-PAGE electrophoresis and transfer to a PVDF membrane. Then membranes were blocked with 5% nonfat milk in TBST buffer before incubation with primary antibodies against MCL-1 (1:1000, Cell Signaling Technology, cat: #94296), BCL-XL (1:1000, Cell Signaling Technology, cat: #2764), cleaved caspase-3 (1:1000, Cell Signaling Technology, cat: #9661), cyclin-D1 (1:1000, Cell Signaling Technology, cat: #55506), p-ERK1/2 (1:1000, Cell Signaling Technology, cat: #4370), t-ERK1/2 (1:1000, Cell Signaling Technology, cat: #4695) and β-actin (1:1000, Cell Signaling Technology, cat: #4970) at 4°C overnight. Secondary antibodies were incubated with TBST-washed membranes for 1 h. After washing for three times, the membranes were reacted with ECL substrate for protein bands detection using chemiluminescent imaging system.

### 2.14 ROS level measurement

Osteosarcoma cells (1 × 10^5^) were seeded onto 6-well plates, incubated overnight. After corresponding Paucatalinone A treatment for 48 h, cells were collected and stained with 10 μM DCFH-DA (Beyotime Biotechnology, cat: S0033S, Ex = 488 nm, Em = 525 nm) for 30 min at 37°C. Cells were washed twice in PBS and the ROS levels were measured by flow cytometry with 1 × 10^4^ events recorded for each sample, and the results were analyzed using FlowJo software.

### 2.15 Intracellular calcium measurement

Intracellular calcium assay kit with Fluo-4 AM fluorescence labeling (Beyotime Biotechnology, cat: S1060, Ex = 488nm, Em = 512–520 nm) was used to detect changes in calcium. Briefly, osteosarcoma cells (1 × 10^5^) were seeded onto 6-well plates, incubated overnight. After treatment with the corresponding Paucatalinone A for 48 h, 2 μM Fluo-4 AM dye was added to the cell suspension and then incubated in the dark for 30 min at 37°C. Then the samples were analyzed using flow cytometry with 1 × 10^4^ events recorded for each sample, and the results were analyzed using FlowJo software.

### 2.16 Caspase-3 assay

GreenNuc™ caspase-3 assay kit for live cells (Beyotime Biotechnology, cat: C1168S, Ex = 500 nm, Em = 530 nm) was used to detect caspase-3 activity. Briefly, osteosarcoma cells (1 × 10^5^) were seeded onto 6-well plates, incubated overnight. After treatment with the corresponding Paucatalinone A for 48 h, single-cell suspension was collected and mixed with 1 μL GreenNuc™ caspase-3 substrate, then incubated for 30 min at 37°C in the dark. Then caspase-3 activity was determined by flow cytometry with 1 × 10^4^ events recorded for each sample, and the results were analyzed using FlowJo software.

### 2.17 Animal study

All the mice used in the study were obtained from Beijing Vital River Laboratory Animal Technology Co., Ltd. All animals were kept in the model animal research centre of Shandong University, and the experimental protocol approved by the ethics committee of the Qilu Hospital of Shandong University (Jinan, China). For subcutaneous inoculation, 6–8 weeks old female BALB/c mice were implanted into the right flank with 1 × 10^6^ K7M2 cells. Once tumor volumes reached 100–150 mm^3^, mice were randomly divided into four groups: normal saline, treated with Paucatalinone A (5 mg/kg), treated with Paucatalinone A (10 mg/kg), treated with cisplatin (10 mg/kg). Paucatalinone A treatment was performed through intravenous (i.v.) administration at the indicated doses every other day for a total of five injections, based on the experimental timeline shown in Figure 7A. Twenty days post-implantation, tumors were excised from mice and weighed.

### 2.18 Statistics

Data were analyzed with GraphPad Prism 8 and presented as mean ± s.d. Statistical analysis for two sets of data was performed using Students’ t-test. For more than two data sets, one-way analysis of variance (ANOVA) was performed. Significance was defined as *p < 0.05, **p < 0.01, ***p < 0.001 and ****p < 0.0001.

## 3 Results

### 3.1 Paucatalinone A showed potent growth inhibition and induced cell cycle arrest in osteosarcoma cells *in vitro*

**FIGURE 2:**
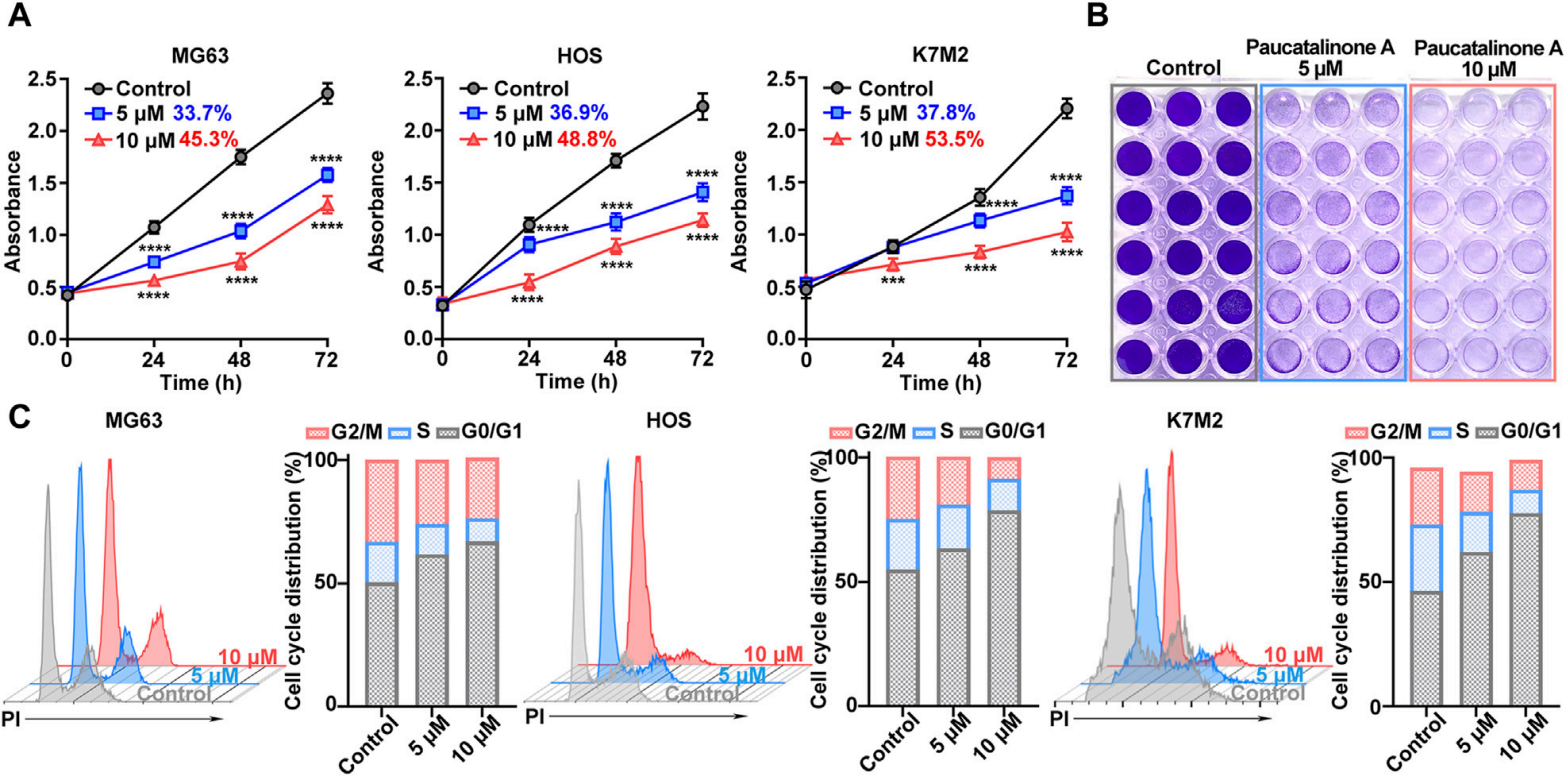
The effect of Paucatalinone A on the proliferation of osteosarcoma cells. **(A)** Cell viability of MG63, HOS and K7M2 treated with Paucatalinone A (5 μM and 10 μM) for 24, 48 and 72 h. The inhibitory rates were shown in numbers (*n* = 6). **(B)** Cell viability assay used crystal violet staining of osteosarcoma cells after different treatment. **(C)** Flow cytometry analysis for cell cycle distribution of MG63, HOS and K7M2 treated with Paucatalinone A (5 μM and 10 μM) (*n* = 3). Data are presented as mean ± s.d. ***p < 0.001, ****p < 0.0001 by Two-way ANOVA.

The effect of Paucatalinone A on cell proliferation in human osteosarcoma cell lines MG63 and HOS, and mouse osteosarcoma cell line K7M2, was evaluated. As shown in Figure 2A and Supplementary Figure S1, it was confirmed that osteosarcoma cells were sensitive to Paucatalinone A, inducing approximately 40% proliferation inhibition of MG63, HOS and K7M2 cells, respectively. And the treatment of the Paucatalinone A significantly inhibited the proliferation of osteosarcoma cells in a dose- and time-dependent manner. Then, we chose Paucatalinone A concentration of 5 μM and 10 μM for further experiments. In Figure 2B, cell viability assay used crystal violet staining showed the inhibitory status of osteosarcoma cells after Paucatalinone A treatment. In addition, we examined whether Paucatalinone A affected osteosarcoma cell growth by altering cell cycle progression. As shown in Figure 2C, cell cycle assessment using flow cytometry analysis revealed that Paucatalinone A arrested the cell cycle in the G0-G1 stage of three osteosarcoma cell lines, accompanied with a decrease in S and G2/M phases, which was in line with the strong proliferation inhibition rates (Figure 2C). In together, these results indicated that Paucatalinone A inhibited the proliferation of osteosarcoma cells by mitigating the G0/G1 transition.

### 3.2 Paucatalinone A induced osteosarcoma cell apoptosis

**FIGURE 3:**
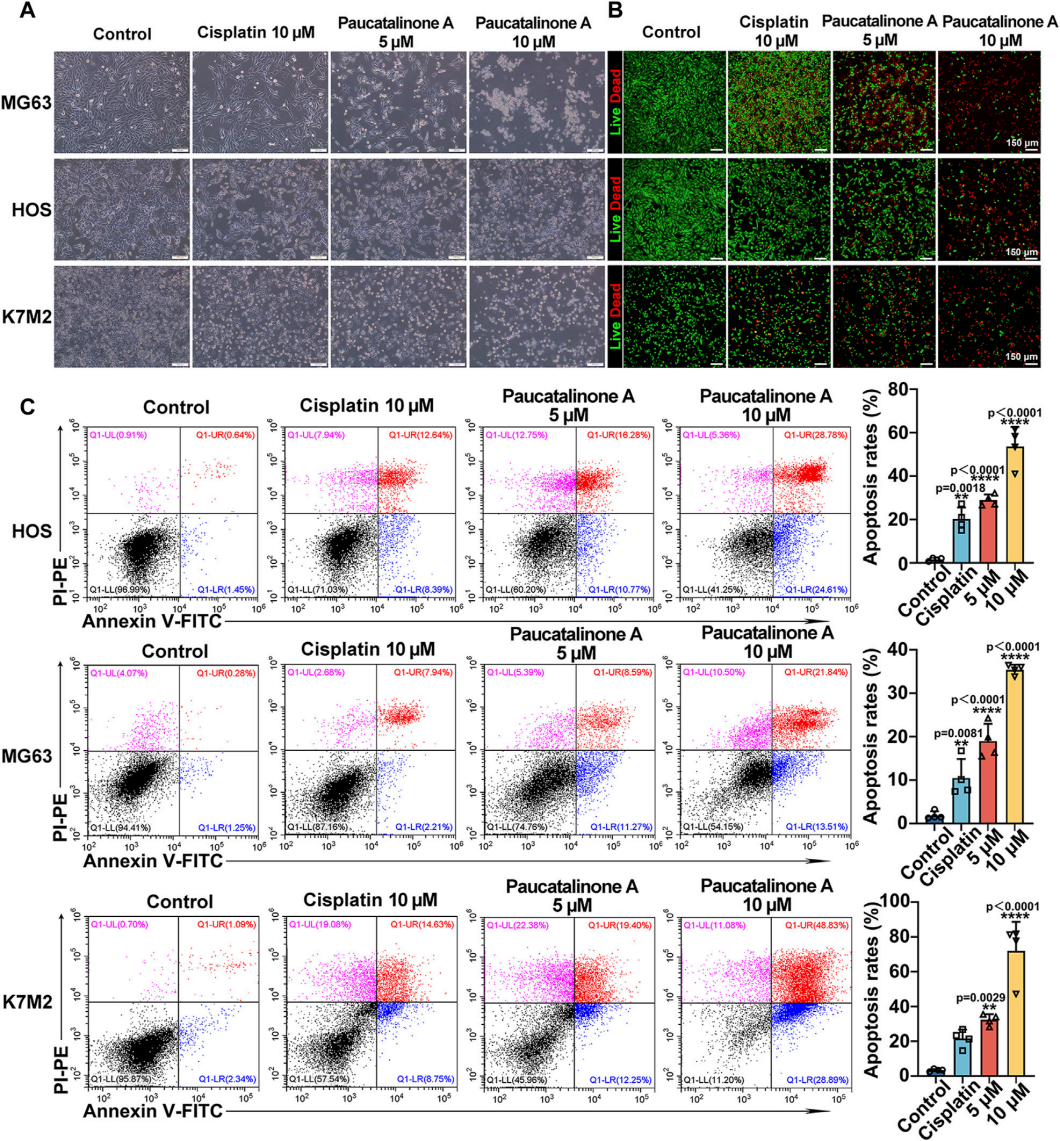
Paucatalinone A induced osteosarcoma cell apoptosis. **(A)** Images of osteosarcoma cells after different treatment. Scale bar indicates 200 μm. **(B)** Fluorescence images of osteosarcoma cells in live/dead staining experiments after Paucatalinone A treatment. Scale bar indicates 150 μm. **(C)** Apoptosis analysis of osteosarcoma cells after different treatment using flow cytometry (*n* = 4). Data are presented as mean ± s.d. **p < 0.01, ****p < 0.0001 by One-way ANOVA.

In order to verify the therapeutic effect of Paucatalinone A in osteosarcoma, we examined the inhibitory status of tumor cells. For the cell viability assay, significant anti-proliferation activity was observed in Paucatalinone A-treated cells, indicating with rounded and retracted morphology (Figure 3A). Moreover, a live/dead viability assay for fluorescence microscopy analysis was performed to reveal the cytotoxicity mechanism. As shown in Figure 3B, Paucatalinone A induced significant death in osteosarcoma cells. To further understand the pro-apoptotic mechanisms of Paucatalinone A in osteosarcoma cells, we analyzed the percent of apoptotic cells after Paucatalinone A treatment using Annexin V/PI cell apoptosis assays by flow cytometry. The results demonstrated a significant increase of Annexin V/PI-positive apoptotic cells with Paucatalinone A treatment, as compared with cisplatin control (Figure 3C). Additionally, we investigated the toxicity of Paucatalinone A to normal cells. Compared with osteosarcoma cells, Paucatalinone A were significantly less toxic to the normal cells (Supplementary Figure S2). Taken together, these results demonstrated that Paucatalinone A could induce apoptosis in osteosarcoma cells.

### 3.3 Paucatalinone A inhibited colony and spheroid formation of osteosarcoma cells

**FIGURE 4:**
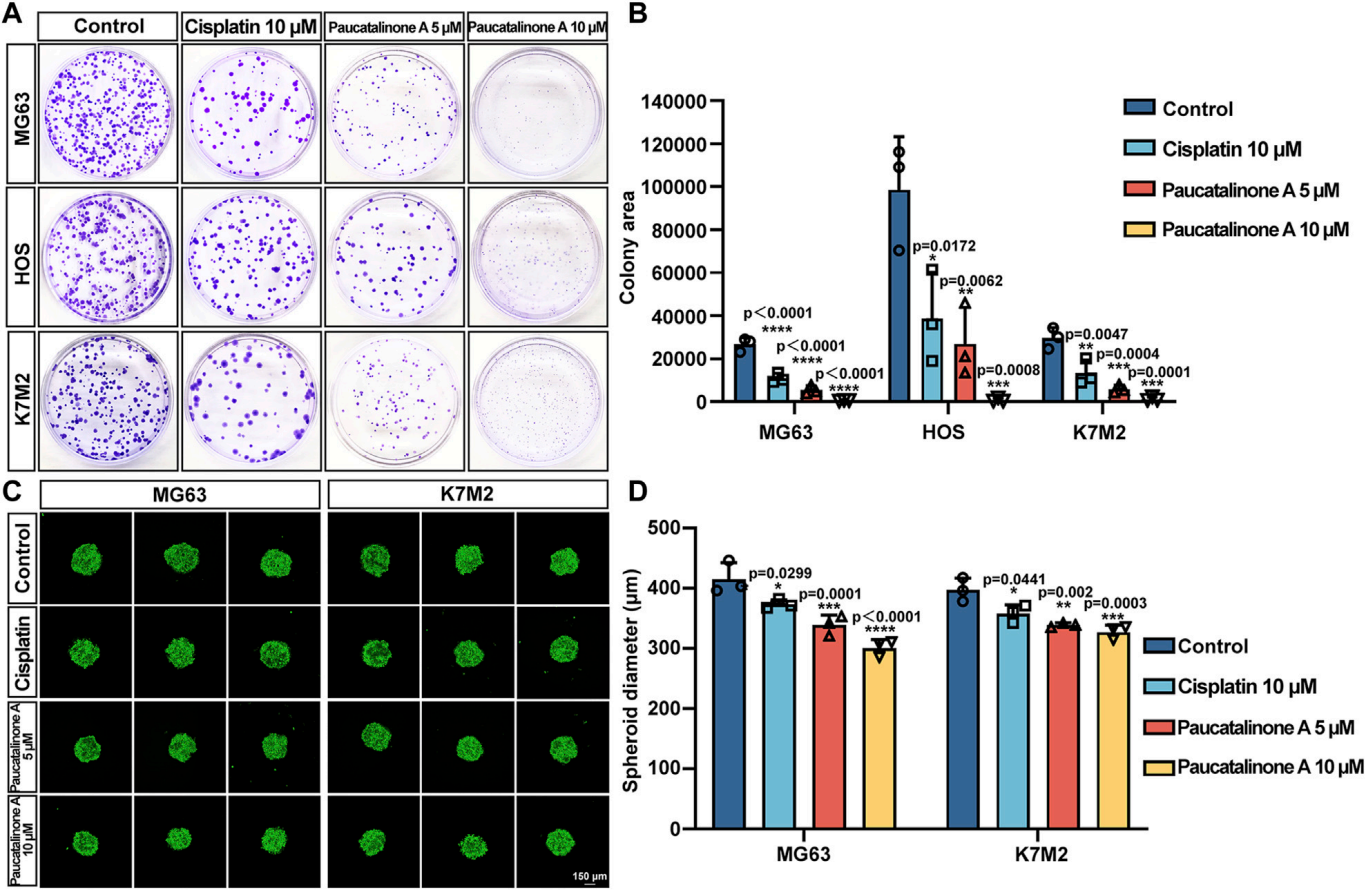
Paucatalinone A suppressed osteosarcoma cell spheroid and colony formation. **(A)** Representative images of colonies in cisplatin and Paucatalinone A treated cells. **(B)** Histogram showing the quantification of the colony area in different groups (*n* = 3). **(C)** Representative confocal images of MG63 (MG63-GFP, green) and K7M2 (K7M2-GFP, green) cell spheroids with diferent treatment. All spheroids were cultured in a 30 µL hanging drop (*n* = 3). Scale bar indicates 150 μm. **(D)** Histogram showing the diameter of MG63 and K7M2 cell spheroids (*n* = 3). Data are presented as mean ± s.d. *p < 0.05, **p < 0.01, ***p < 0.001, ****p < 0.0001 by One-way ANOVA.

Having prompted by above observations, we decided to explore the ability of cell-cell cohesion and single cell viability. Tumor cells could colonize in distant organs in tumor progression and metastasis (Fares et al., 2020). We examined the effect of Paucatalinone A on colony formation ability of osteosarcoma cells. As shown in Figures 4A, B, the number and size of colonies decreased in Paucatalinone A-treated cells. Compared with traditional monolayer cell culture, the three-dimensional (3D) tumor spheroid has emerged as an essential *in vitro* model for cancer research due to the recapitulation of the architecture and physiology of solid human tumors. Herein, we used a 3D tumor spheroid culture experiment to evaluate the proliferation inhibiting function of Paucatalinone A to osteosarcoma cells. As shown in Figures 4C, D, Paucatalinone A treatment significantly decreased the diameter of the 3D tumor spheroids. In together, the results indicated that Paucatalinone A inhibited osteosarcoma colony formation and tumor spheroid ability in a dose-dependent fashion.

### 3.4 Paucatalinone A retarded osteosarcoma cell migration and disrupted the microfilaments

**FIGURE 5:**
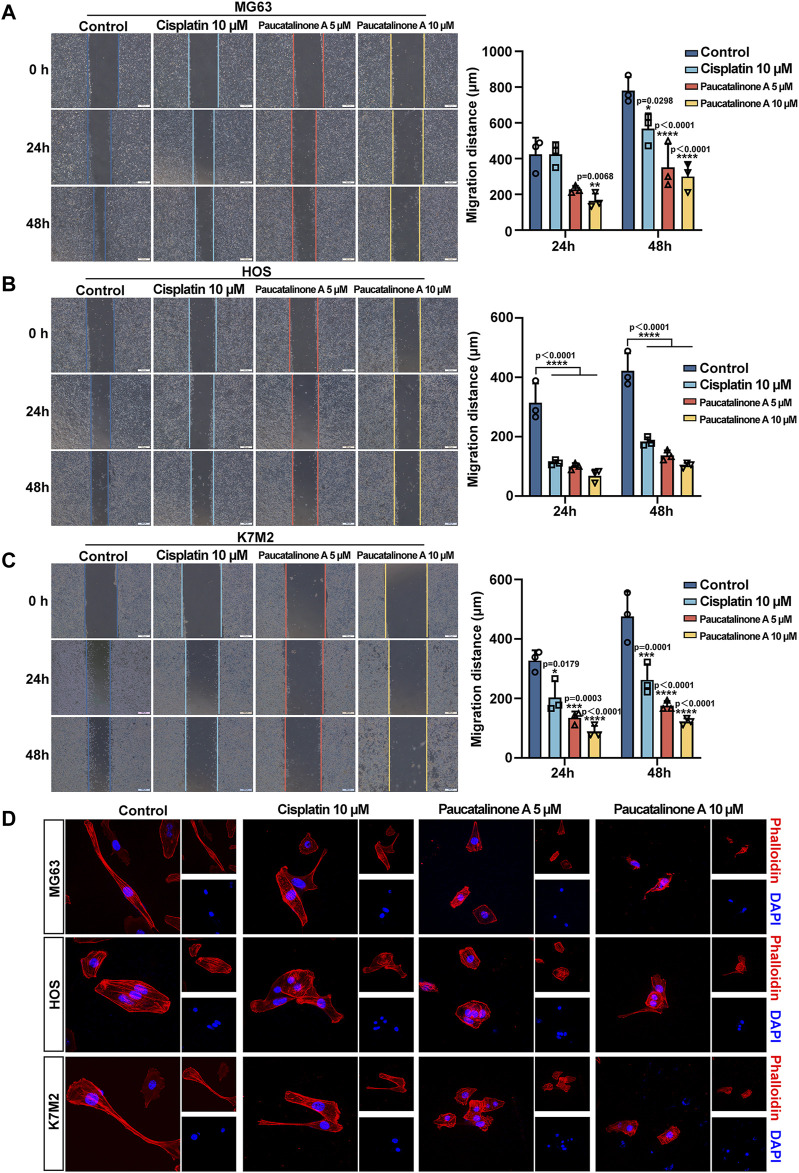
Paucatalinone A inhibited the migration of osteosarcoma cells. **(A–C)** Migration of MG63, HOS and K7M2 cells were evaluated using the *in vitro* wound-healing scratch assay. The quantitative analysis showing the migration distance of cells (*n* = 3). Scale bar indicates 500 μm. **(D)** Confocal analysis showing the cellular F-actin stained by phalloidin after different treatment. Scale bar indicates 150 μm. Data are presented as mean ± s.d. *p < 0.05, **p < 0.01, ***p < 0.001, ****p < 0.0001 by One-way ANOVA.

Cell migration and invasion was the early process of cancer metastatic cascade (Wells et al., 2013). We further examined whether Paucatalinone A treatment block the properties of migration of osteosarcoma cells. First, we utilized a classic wound-healing model to assess the cell migration ability. As shown in Figures 5A–C, Paucatalinone A treatment markedly inhibited osteosarcoma cell migration compared with control groups. The cytoskeleton is a network of filaments and tubules that extends throughout a cell, which can support the cell, give it shape, organize and tether the organelles, and have roles in molecule transport, cell division and cell signaling (Seetharaman and Etienne-Manneville, 2020). Therefore, we explored the F-actin architecture in Paucatalinone A-treated cells staining with conjugated phalloidin-TRITC by confocal microscopy. As shown in Figure 5D, Paucatalinone A significantly disrupted actin microfilaments in osteosarcoma cells, indicating that Paucatalinone A induced actin depolymerization. Together, these results indicated that Paucatalinone A could efficiently disordered microfilaments of osteosarcoma cells, thus preventing cell migration activity.

### 3.5 Paucatalinone A induced osteosarcoma cell apoptosis via intrinsic apoptotic pathway

**FIGURE 6:**
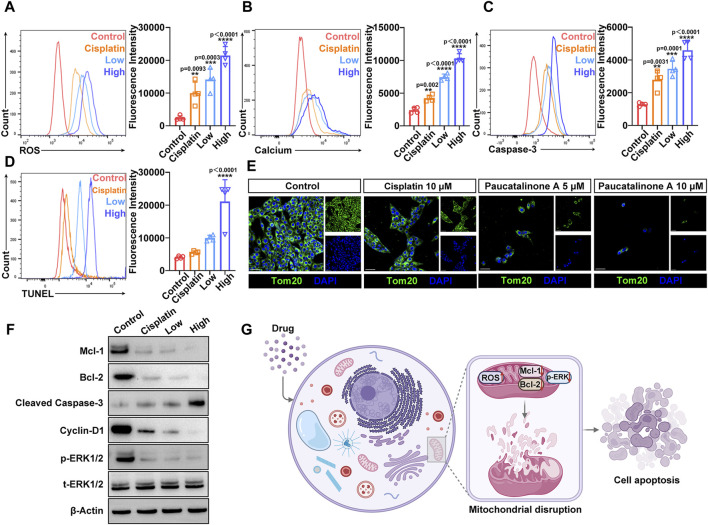
Paucatalinone A induced osteosarcoma cell apoptosis mainly by mitochondrial destruction. **(A)** Flow cytometry analysis and fluorescence quantification of ROS level in osteosarcoma cells (*n* = 4). **(B)** Flow cytometry analysis and fluorescence quantification of intracellular calcium level in osteosarcoma cells (*n* = 4). **(C)** Flow cytometry analysis and fluorescence quantification of cleaved caspase-3 level in osteosarcoma cells (*n* = 4). **(D)** Flow cytometry analysis and fluorescence quantification of TUNEL level in osteosarcoma cells (*n* = 4). **(E)** Confocal images of staining for the mitochondrial structural protein Tom-20 after different treatments. Scale bar indicates 40 μm. **(F)** Western blot showing Mcl-1, Bcl-2, Cyclin D1, p-ERK1/2 and t-ERK1/2 protein expression levels in osteosarcoma cells after different treatments. **(G)** Schematic illustration of the Paucatalinone A destroying mitochondria that lead to osteosarcoma cell apoptosis. Data are presented as mean ± s.d. **p < 0.01, ***p < 0.001, ****p < 0.0001 by One-way ANOVA.

Accumulation of reactive oxygen species (ROS) is closely associated with the activation of intrinsic apoptotic pathways (Cheung and Vousden, 2022). To investigate this, we measured intracellular ROS levels in osteosarcoma cells following treatment with Paucatalinone A, utilizing DCFH-DA (Dichlorodihydrofluorescein diacetate) staining and flow cytometry for detection. Given that mitochondria are the primary source of ROS in cells, our findings, as illustrated in Figure 6A, indicate that Paucatalinone A significantly increases intracellular ROS levels in a dose-dependent manner. In addition to ROS, intracellular free calcium acts as a critical second messenger in regulating various physiological processes. We subsequently examined changes in intracellular free Ca^2+^ levels following Paucatalinone A treatment in osteosarcoma cells. As shown in Figure 6B, after Fluo-4/AM staining, the osteosarcoma cells exhibited a higher Ca^2+^ levels with Paucatalinone A treatment. The activation of the caspase cascade is a hallmark of apoptosis. To further explore this, we quantified the levels of cleaved caspase-3 in osteosarcoma cells treated with Paucatalinone A. Our results reveal a significant increase in cleaved caspase-3 levels in cells exposed to Paucatalinone A (Figure 6C), indicating activation of apoptotic pathways. Additionally, TUNEL assays were conducted to assess DNA damage, further supporting the pro-apoptotic effect of Paucatalinone A on osteosarcoma cells. Figure 6D shows that treatment with Paucatalinone A induces notable DNA damage in these cells, corroborating the induction of apoptosis through intrinsic pathways.

Mitochondria are involved in many cell activities. Based on the above results, we hypothesized that Paucatalinone A induced osteosarcoma cell apoptosis through mitochondrial disruption. Tom-20 is located at the outer membrane of mitochondria to facilitate the transportation of proteins across mitochondrial membrane, and plays vital role in the executive function of mitochondria (Su et al., 2022). We further examined the Tom-20 expression in osteosarcoma cells treated with Paucatalinone A through confocal microscopy. As shown in Figure 6E, the data suggested that Paucatalinone A significantly reduced Tom-20 expression, indicating that the drug could strongly damage the mitochondria of osteosarcoma cells. Moreover, to elucidate the intrinsic mechanisms underlying the anticancer effect of Paucatalinone A, we sought to explore the relevant signaling pathway. As shown in Figure 6F, Paucatalinone A also decreased the expression of Mcl-1, Bcl-2 and elevated cleaved caspase-3 levels in tumor cells, which were involved in mitochondria-mediated apoptosis. The cyclin D1 and ERK1/2 signaling pathway controls various primary cellular processes such as cell survival, proliferation, fate determination, and stress responses. Aberrant ERK1/2 signaling underlies a wide range of disorders in humans, such as aging and cancer. In the present study, we revealed that cyclin D1 and p-ERK1/2 protein expression was significantly decreased by treatment with Paucatalinone A (Figure 6F). Taken together, our results revealed that Paucatalinone A could induce the apoptosis of osteosarcoma cells by disturbing the mitochondria/ERK1/2 signaling pathway (Figure 6G).

### 3.6 Paucatalinone A indicated antitumor efficacy in osteosarcoma mouse model

**FIGURE 7:**
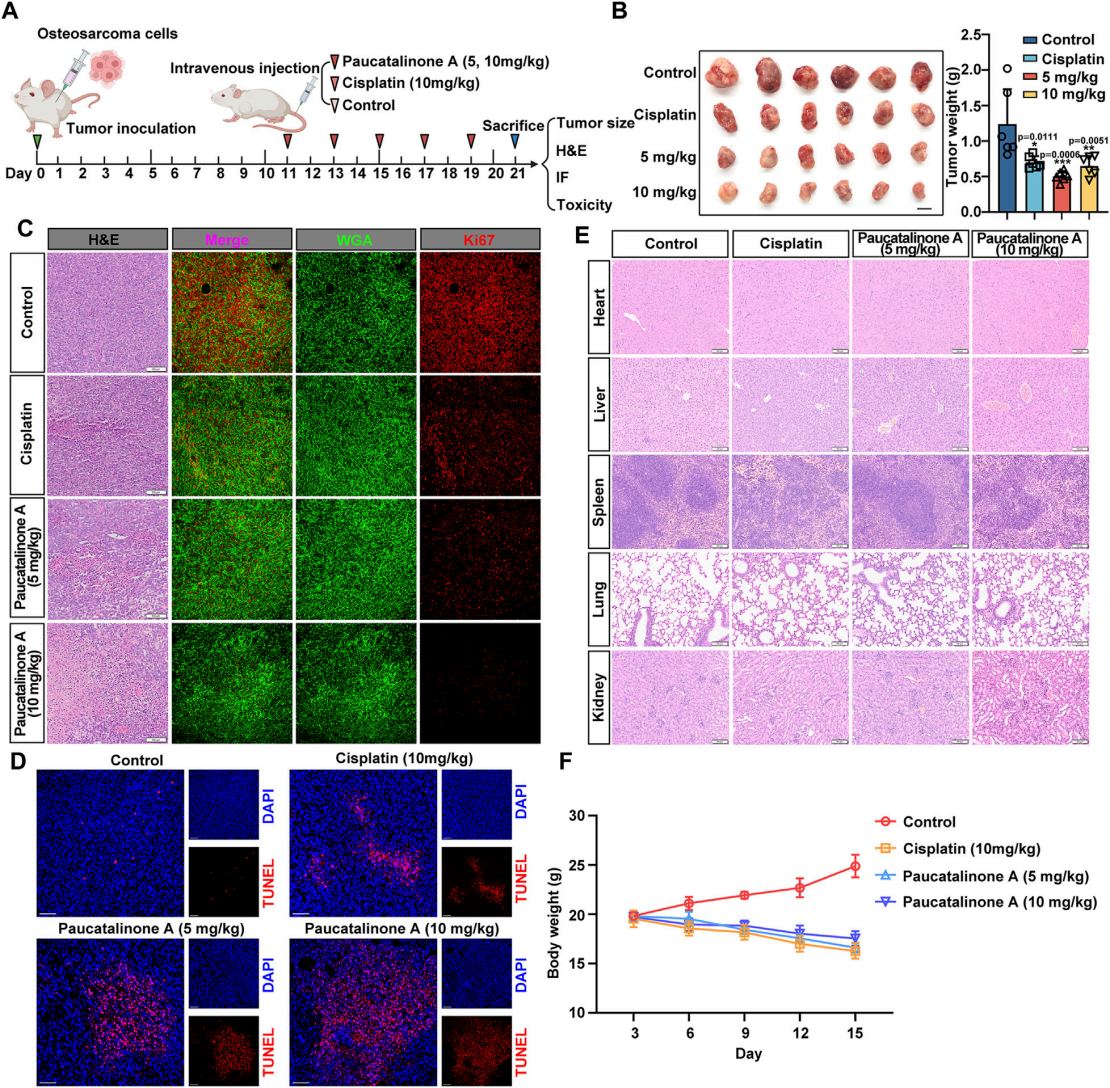
Paucatalinone A inhibited the growth of osteosarcoma tumor in mice. **(A)** Diagram showing the experimental procedure *in vivo*. **(B)** Images of osteosarcoma tumors harvested from mice with different treatment (*n* = 6). Scale bar indicates 0.5 cm. Statistical diagram showing the tumor weight of different groups (*n* = 6). **(C)** Images showing H&E-staining slides and immunofluorescence which red signal indicates Ki67 and green signal indicates WGA. Scale bars in H&E staining represent 100 μm and immunofluorescence images represent 10 μm. **(D)** Immunofluorescence images showing TUNEL staining of osteosarcoma tumors. Scale bar indicates 150 μm. **(E)** Representative H&E staining images of heart, liver, spleen, lung and kidney from mice with cisplatin and Paucatalinone A treatment. **(F)** Body weight analysis from mice with different treatment (*n* = 6). Data are presented as mean ± s.d. *p < 0.05, **p < 0.01, ***p < 0.001 by One-way ANOVA.

Based on the encouraging results that Paucatalinone A induced osteosarcoma cell apoptosis by mitochondrial disruption, we further explored the therapeutic efficacy of Paucatalinone A on osteosarcoma mouse model *in vivo*. As shown in Figure 7A, mice were treated with different doses of Paucatalinone A while cisplatin was used as positive control after tumor establishment. Throughout the study, tumor appearance was observed on day 10, and the tumor size was evaluated until the day on which the mice were sacrificed. The results showed that treatment with Paucatalinone A (10 mg/kg) inhibited tumor growth and decreased tumor weight (Figure 7B). In addition, the results of Ki67 staining of tumor sections showed that Ki67-positive cells of mouse osteosarcoma tumors in high-dose Paucatalinone A treatment group were the lowest, indicating that the proliferation of osteosarcoma was dramatically inhibited (Figure 7C).

To further verified the apoptosis induced effect of Paucatalinone A in tumor model, we performed in terminal deoxynucleotidyl-transferasemediated dUTP nick-end labeling (TUNEL)-stained osteosarcoma tumor sections, the most severe apoptosis was detected in the Paucatalinone A (10 mg/kg) treatment group (Figure 7D). Together, these results demonstrated that Paucatalinone A exhibited superior antitumor activity in osteosarcoma mouse model. In addition, H&E staining showed that there were no significant pathological changes in the vital organs (heart, liver, spleen, lung, kidney) of the mice with different drugs treatment compared with the mice from control group (Figure 7E). Similarly, it is worth mentioning that during the period of drug treatment, these mice did not display significant signs of illness or weight loss (Figure 7F). In conclusion, both of these results preliminarily revealed the good biocompatibility of the Paucatalinone A and validated that the toxicity of the Paucatalinone A is tolerable.

## 4 Discussion

Recent advances made in osteosarcoma treatment are revolutionary, which can bring us to an inflection point in the trajectory of translational and clinical osteosarcoma research that will lead to continued improvements in survival. Young adults with resectable osteosarcoma are assigned to treatment with high-dose methotrexate, doxorubicin, and cisplatin (MAP) chemotherapy regimen (Marina et al., 2016; Sayles et al., 2019). High-dose chemotherapy is frequently avoided in the treatment of older patients due to the increased risk of long-term toxic effects in this age group. Numerous studies have sought to identify new agents that could potentially replace the methotrexate, Adriamycin (doxorubicin), and cisplatin (MAP) regimen. However, to date, none of these alternative regimens have demonstrated superiority over traditional chemotherapeutic strategies in patients newly diagnosed with osteosarcoma.

With the advancement of information technique and bioinformatics, there is an increasing trend to build resources and databases that report natural compounds, active components of the plant, and related information (Rodrigues et al., 2016; Mazumder et al., 2018). Although plant-based compounds have shown be less toxic compared to conventional synthetic compounds, there is growing evidence on the side effects of the unregulated use of these plants against different diseases (Cech and Oberlies, 2023). The problem is that there is insufficient data available regarding the quality, safety, and efficacy of these compounds. Globally, the process of oncology drug development and marketing is regulated through the involvement of experts and an advisory process mediated by regulatory authorities. Considering the fact that plant-based drug formulations usually consist of several phytochemicals or even more than one plants. The major challenge on this direction would be to predict the role of phytochemicals in traditional medicine. In our study, to illustrate the anticancer property of Paucatalinone A isolated from *Paulownia Catalpifolia* Gong Tong, we put forward that Paucatalinone A played a crucial role in the bioactive effect of the plant. We performed both *in vitro* and *in vivo* studies to revealed that Paucatalinone A possessed biological effect in osteosarcoma progression.

**FIGURE 8:**
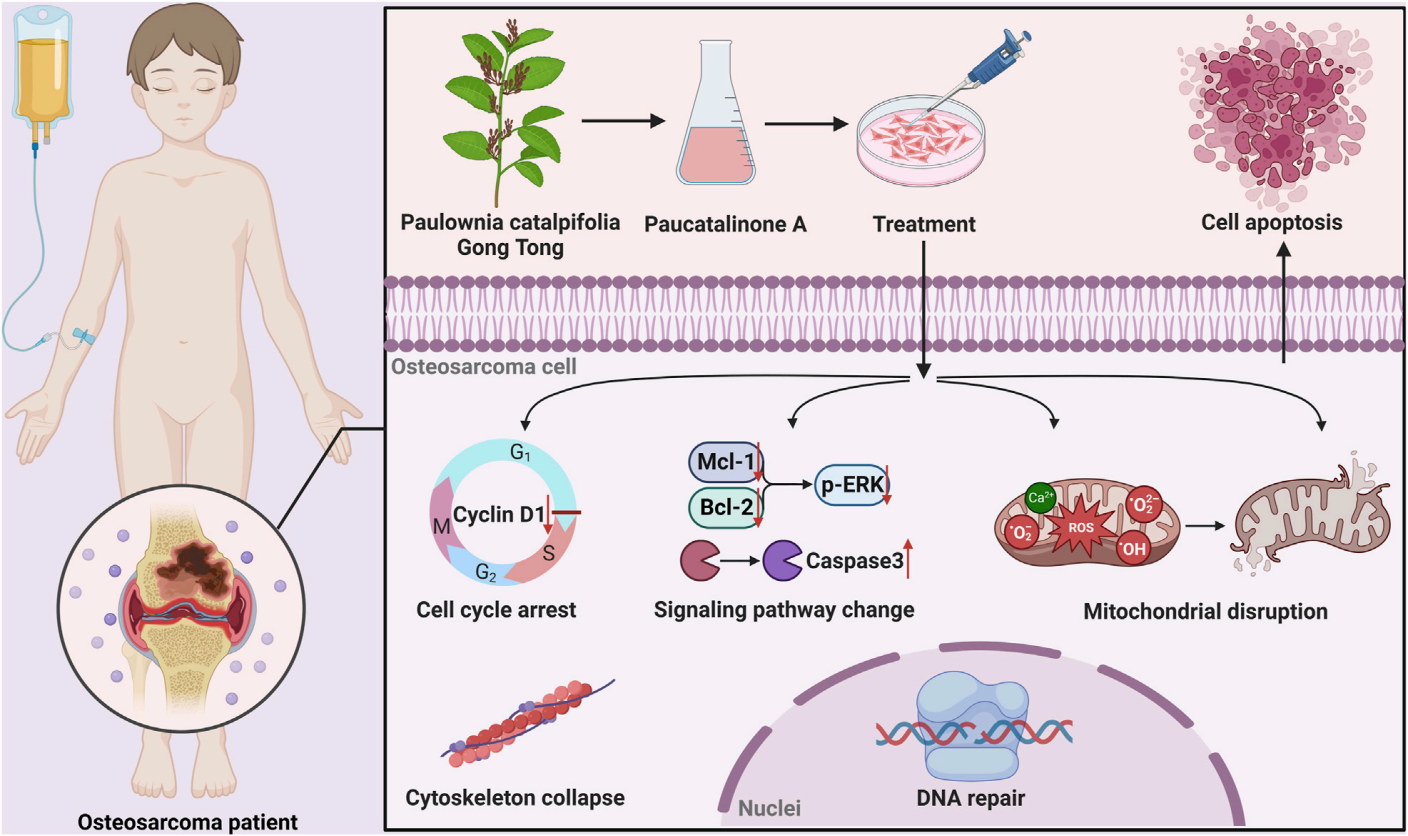
Graphical abstract showed that Paucatalinone A dramatically induced osteosarcoma cell apoptosis through intrinsic mitochondrial pathway, which might provide a feasible therapy for osteosarcoma.

Apoptotic cell death is a major form of regulated cell death and plays a central role in the development and homeostasis of cellular organisms (Bedoui et al., 2020). Insufficient or suppression of apoptosis is a hallmark of cancer and can lead to severe pathological consequences (Mohammad et al., 2015). Mitochondria acts as the power of life, and are crucial for the initiation of apoptosis (Burke, 2017; Harrington et al., 2023). Upon the induction of mitochondrial apoptosis (such as ROS generation, DNA damage), mitochondrial outer membrane permeabilization (MOMP) usually commits, resulting in apoptotic downstream signalling activation, involves cytochrome c release and caspase activation (Bock and Tait, 2020; Winter et al., 2022). Caspases are typically recognized as specific protease. Once activated, the effector caspases are responsible for proteolytic cleavage of substrates, resulting ultimately to cell death. Previous research has demonstrated that chemical analogues of Paucatalinone A possess anti-tumor properties, primarily through the induction of endogenous apoptosis in tumor cells (Smejkal et al., 2007; Smejkal et al., 2010; Hanáková et al., 2015; Xiao et al., 2023). In this study, we demonstrated that Paucatalinone A induced tumor cell mitochondria disruption mediated by anti-apoptotic proteins (Bcl-2, Mcl-1), pro-apoptotic family member ERK1/2, or calcium and ROS-triggered mitochondrial permeability transition. Taken together, these findings have placed the mitochondria in the focus of current cell death research, and therapeutic targeting of mitochondrial apoptosis has great clinical potential in various diseases (Figure 8).

In summary, we elaborated Paucatalinone A, a newly geranylated flavanones isolated from *Paulownia Catalpifolia* Gong Tong, for cascade cell apoptotic modulation to combat osteosarcoma. We found that Paucatalinone A dramatically induced tumor cell apoptosis through intrinsic mitochondrial pathway. Meanwhile, Paucatalinone A-based therapy exhibited superior antitumor effects in osteosarcoma mouse model and ultimately lead to an enhanced tumor inhibition. Overall, our study combined *in vivo* and *in vitro* experiment to provide insights on the molecular mechanisms involved in osteosarcoma, and we showed the efficacy of Paucatalinone A as a new therapeutic candidate on malignancies in future.

## Data Availability

The original contributions presented in the study are included in the article/Supplementary Material, further inquiries can be directed to the corresponding authors.
