# Supplementary material for: Paucatalinone A from Paulownia Catalpifolia Gong Tong Elicits mitochondrial-mediated cancer cell death to combat osteosarcoma
Source: Front Pharmacol. 2024 Mar 25;15:1367316. doi: 10.3389/fphar.2024.1367316 (PMC10999585; doi:10.3389/fphar.2024.1367316)
Supplement: Supplementary file 1 [file DataSheet1.PDF]

Supplementary Material

Paucatalinone A from Paulownia Catalpifolia Gong Tong Elicits Mitochondrial-Mediated Cancer Cell Death to Combat Osteosarcoma

Ganyu Wang[1], Zhiwei Cui[2], Jinqiu Tian[1], Xinyuan Li[3], Wenzhao Tang[4], Weiqiang Jing[5], Aiwu Li[1*], Yuankai Zhang[2*]

[1]Department of Pediatric Surgery, Qilu Hospital, Cheeloo College of Medicine, Shandong University, Jinan, Shandong Province 250012, China

[2]Department of orthopedics, Qilu Hospital, Cheeloo College of Medicine, Shandong University, Jinan, Shandong Province, 250012, China

[3]Department of Immunology, Shandong Provincial Key Laboratory of Infection & Immunology, School of Basic Medical Sciences, Cheeloo College of Medicine, Shandong University, Jinan, Shandong Province, 250012, China

[4]School of Parmacy and Pharmaceutical Sciences & Institute of Materia Medica, Shandong First Medical University & Shandong Academy of Medical Sciences, NHC Key Laboratory of Biotechnology Drugs (Shandong Academy of Medical Sciences), Key Lab for Rare & Uncommon Diseases of Shandong Province, Jinan 250117, China

[5]Department of Urology, Qilu Hospital, Cheeloo College of Medicine, Shandong University, Jinan, Shandong Province 250012, China

*** Correspondence:**
Yuankai Zhang, M.D. Email: drzhangyk@163.com; Aiwu Li, M.D. Email: liaiwu@qiluhospital.com.

Supplementary Figures and Figure Legends

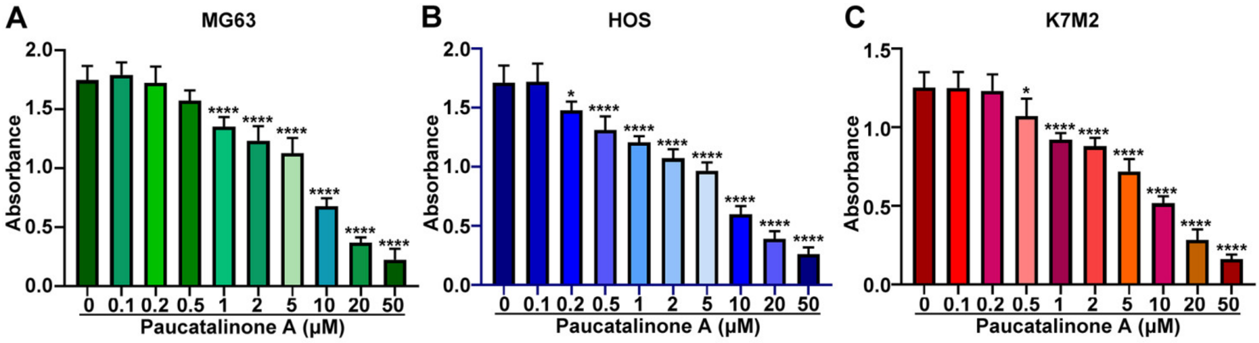

Supplementary Figure 1. Cell viability of MG63 **(A)**, HOS **(B)** and K7M2 **(C)** treated with different doses of Paucatalinone A for 72 h (n=4). Data are presented as mean ± s.d. *$p < 0.05$, ****$p < 0.0001$ by One-way ANOVA.

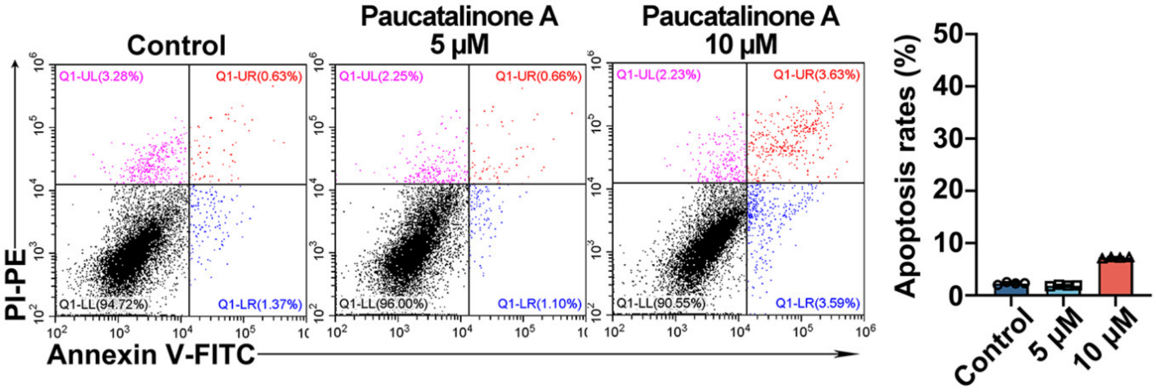

Supplementary Figure 2. Apoptosis analysis of HUVEC cells after different treatment using flow cytometry (n=4). Data are presented as mean ± s.d.
